# Immunological Pathogenesis of Membranous Nephropathy: Focus on PLA2R1 and Its Role

**DOI:** 10.3389/fimmu.2019.01809

**Published:** 2019-08-06

**Authors:** Wenbin Liu, Chang Gao, Haoran Dai, Yang Zheng, Zhaocheng Dong, Yu Gao, Fei Liu, Zihan Zhang, Zhiyuan Liu, Weijing Liu, Baoli Liu, Qingquan Liu, Jialan Shi

**Affiliations:** ^1^Beijing Hospital of Traditional Chinese Medicine, Capital Medical University, Beijing, China; ^2^Capital Medical University, Beijing, China; ^3^Beijing University of Chinese Medicine, Beijing, China; ^4^Shunyi Branch, Beijing Hospital of Traditional Chinese Medicine, Beijing, China; ^5^Taishan Medical University, Shandong, China; ^6^Key Laboratory of Chinese Internal Medicine of Ministry of Education, Beijing, China; ^7^Departments of Medicine, VA Boston Healthcare System, Brigham and Women's Hospital, Harvard Medical School, Boston, MA, United States

**Keywords:** membranous nephropathy, immunological pathogenesis, PLA2R1, PM2.5, lung, kidney

## Abstract

Membranous nephropathy (MN) is the major cause of nephrotic syndrome with special pathological features, caused by the formation of immune complexes in the space between podocytes and the glomerular basement membrane. In idiopathic membranous nephropathy (IMN) the immune complexes are formed by circulating antibodies binding mainly to one of two naturally-expressed podocyte antigens: the M-type receptor for secretory phospholipase A2 (PLA2R1) and the Thrombospondin type-1 domain-containing 7A (THSD7A). Formation of antibodies against PLA2R1 is much more common, accounting for 70–80% of IMN. However, the mechanism of anti-podocyte antibody production in IMN is still unclear. In this review, we emphasize that the exposure of PLA2R1 is critical for triggering the pathogenesis of PLA2R1-associated MN, and propose the potential association between inflammation, pollution and PLA2R1. Our review aims to clarify the current research of these precipitating factors in a way that may suggest future directions for discovering the pathogenesis of MN, leading to additional therapeutic targets and strategies for the prevention and early treatment of MN.

## Introduction

Membranous nephropathy (MN) is an immune-mediated glomerular disease that affects 12 new cases out of every million adults every year globally (1, 2). MN is characterized by a pathological change in the glomerular basement membrane (GBM) caused by the accumulation of immune complexes which appear as granular deposits of immunoglobulin (Ig)G when imaged with immunofluorescence and as electron-dense deposits under electron microscopy. These immune deposits in the space between podocytes and GBM contain the complement membrane attack complex (C5b-9). MN can be either idiopathic (idiopathic membranous nephropathy or IMN) or caused partially by clinical disease such as hepatitis B, systemic lupus erythematosus, cancer, or drug side-effect (secondary membranous nephropathy or SMN) (1). However, it is not clear whether these clinical diseases are direct causes of MN or merely triggers, such as the potential association between cancer and MN (3–5). In addition to the typical GBM changes, SMN exhibits mesangial deposits, “full-house” immunostaining (IgG, IgA, IgM, C3, and C1q, mesangial deposits, and reticular aggregates) in membranous lupus nephritis, and amyloid or fibrillary glomerulonephritis which are seen as fibrils under electron microscopy (Table 1) (6). The deposition of immune complexes in SMN may be caused by exogenous antigens “pre-planted” between podocytes and the GBM which bind to circulating antibodies, such as in hepatitis B virus-associated MN (7). The formation of glomeruli subepithelial immune complex deposits in IMN is now believed to be mediated by specific intrinsic podocyte antigens and their corresponding autoantibodies in humans, such as neutral endopeptidase (NEP), M-type receptor for secretory phospholipase A2 (PLA2R1), and Thrombospondin type-1 domain-containing 7A (THSD7A) (8–10). PLA2R1 (70–80% of IMN) and THSD7A (3–5% of IMN), the two major podocyte antigens identified in adult IMN, can be detected through both direct immunofluorescence staining of renal tissue and detection of their autoantibodies in serum for diagnosis and prognosis (1).

**Table 1:** Pathogenic factors, histopathological features, and clinical outcomes of secondary and idiopathic membranous nephropathy.

	**Secondary MN**	**Idiopathic MN**
Pathogenic factors	**Immune diseases:** Systemic lupus erythematosus, rheumatoid arthritis, hashimoto thyroiditis, sjögren's syndrome, psoriasis, sarcoidosis, mixed connective tissue disease, IgG4-related disease.	**Genetic predisposition:** Two risk alleles (HLA-DQA1 and PLA2R1) were identified in patients with IMN using the genome wide association study of single nucleotide polymorphism (SNP) genes.
	**Infections:** Hepatitis B virus, hepatitis C virus, syphilis, schistosomiasis, HIV, helicobacter pylori, streptococcal infection, malaria, leprosy.	
	**Drugs and toxins:** Penicillamine, tiopronin, gold, mercury, lithium, formaldehyde, NSAIDs, captopril, clopidogrel.	
	**Tumors:** Various solid tumors and lymphomas.	
	**Miscellaneous:** Diabetes mellitus, renal transplantation, sickle cell disease, haematopoietic stem cells transplantation.	
Histopathological features	**Light microscopy:** Progressive homogeneous thickening of the capillary wall and significant mesangial proliferation.	**Light microscopy:** Progressive homogeneous thickening of the capillary wall.
	**Immunofluorescence:** Positive staining for IgG1 or IgG3, IgA, IgM; positive staining for C1q, C3, C4; subepithelial and mesangial immunofluorescence deposition.	**Immunofluorescence:** Positive staining for Mostly IgG4, relatively little IgG1; positive staining for C3, C4, rarely C1q; subepithelial immunofluorescence deposition.
	**Electron microscopy:** Electron-dense deposits in subepithelial, intramembranous, mesangial and tubuloreticular inclusions.	**Electron microscopy:** Electron-dense deposits in subepithelial and intramembranous.
Clinical outcome	Clinically induced by drugs or toxins, usually followed by spontaneous remission after pathogen withdrawal. Therefore, detailed understanding of the patient's medical history is important.	Spontaneous remission occurs in up to 30–40% of cases; the remaining two-thirds of the patients present with persistent proteinuria, and ~40% of those will progress to ESRD within 10 years.

*MN, Membranous nephropathy; HIV, Human Immunodeficiency Virus; HLA-DQA1, The gene encoding HLA complex class II HLA-DQ alpha chain 1(SNP rs2187668); PLA2R1, The gene encoding M-type phospholipase A2 receptor (SNP rs4664308); SNP, Single-nucleotide polymorphisms; IgG, immunoglobulin G; IgA, immunoglobulin A; IgM, immunoglobulin M; C1q, C3, and C4 are all complement components; ESRD, End-stage renal disease*.

In recent years in China, the morbidity of MN has been gradually increasing possibly due to long-term exposure to air pollution, mainly fine particulate matter of <2.5 μm (PM2.5). Each 10 mg/m^3^ increase in PM2.5 concentration over 70 mg/m^3^ is associated with 14% higher odds for MN (11). However, the association between the air pollution and the pathogenesis of MN remains unclear. It is worth noting that PLA2R1 and THSD7A are naturally expressed in other parts of the human body, such as the lungs (3). In this review, we have focused on the possible mechanisms of anti-podocyte antibody production, particularly in PLA2R1-associated MN, and proposed several hypotheses, which may be beneficial to further exploration of the pathogenesis of IMN.

## The Definition and Pathology of Membranous Nephropathy

**Figure 1:**
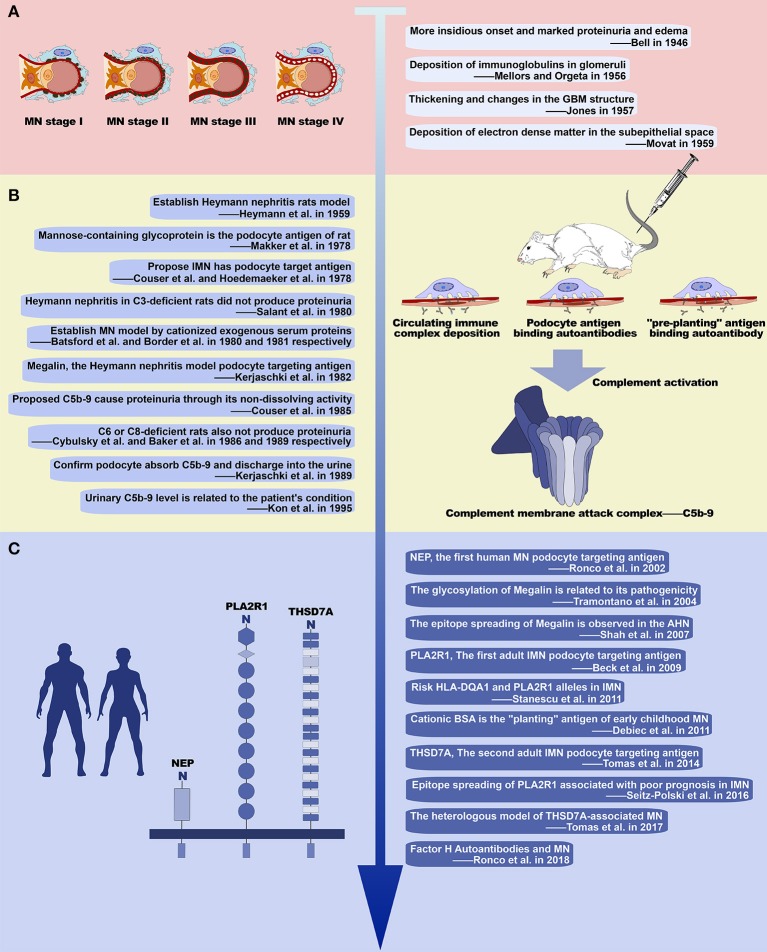
History of the study of membranous nephropathy. **(A)** Basic pathological features of MN. Illustration of the progression of glomerular lesions in MN (left) with four early studies describing its basic pathological features (right). **(B)** Studies of experimental MN and important results. Ten seminal studies of experimental MN (left) and the major discoveries relating to development of MN, including immune complex deposition and complement activation (right). **(C)** Recent advances in our understanding of MN. Three major podocyte antigens found in human MN (left), and other major work on MN since 2000 (right).

Membranous nephropathy, originally called membranous glomerulonephritis (MGN), was first described in 1946 by Bell as a type of glomerular disease, which manifests pathologically as a thickening of the GBM and is clinically characterized by marked proteinuria and edema (12). In the later half of the 1950s, understanding of the pathology of MN developed rapidly, driven by several outstanding pioneers. In 1956, Mellors and Orgeta discovered immunoglobulins containing in the glomeruli deposits using immunofluorescence. In 1957, Jones demonstrated the presence of silver-positive rods projecting from the GBM using periodic acid silver methenamine stain. And in 1959, Movat defined the causal relationship between thickening of the GBM and protein deposition between the GBM and the podocyte using electron microscopy (12). In this way, these pioneers identified the basic features of glomerular lesions in MN, consisting of changes in GBM structure caused by subepithelial electron-dense deposits (Figure 1A). Moreover, in 1968, Ehrenreich used repeated renal biopsy to describe the four stages of glomerular lesions in MN: Stage I consists of only a few small subepithelial deposits such that the GBM may appear normal or be slightly thickened under light microscopy; in stage II spikes protruding from the GBM can be observed using appropriate staining; in stage III the deposits are incorporated within the GBM; in stage IV the GBM appears to be irregularly thickened by reabsorbed deposits, and in complete clinical remission the deposits may disappear and leave some areas lucent or the GBM may return to normal (13) (Figure 1A). These pathological discoveries in the middle of the last century not only defined MN as a unique type of renal pathology, but also guided the development of clinical practices that are still in use today (6).

## Enlightenment Brought by Heymann Nephritis

### Megalin, the Rat Podocyte Antigen

In 1959, Heymann et al. established an animal model that develops pathological manifestations similar to human MN by immunizing rats with tissue components of the proximal tubular brush border (Heymann nephritis or HN) (Figure 1B) (14). This animal model relies on the active immune response of the rat and therefore is often referred to as active Heymann nephritis (AHN). In passive Heymann nephritis (PHN), an analogous lesion can be induced more rapidly by administering heterologous antibody to tubular brush border (anti-Fx1A antibody) (15). At the time, it was thought that the deposition of subepithelial immune complex was caused by circulating immune complexes because Fx1A and its antibody are simultaneously present in glomerular deposits and circulation in HN (16, 17), and the development of immune complexes in circulation was found to be coincident with the glomerular deposits (18). However, in 1978, Couser et al. and Hoedemaeker et al. simultaneously induced HN pathological changes in isolated rat kidneys using anti-Fx1A antibodies suggesting that IMN is not caused by the deposition of circulating immune complexes, but in instead by *in situ* immune complex formed by the autoantibodies binding to antigens located on podocytes (19, 20). In the same year, Makker et al. further confirmed that the podocyte antigen in HN is a mannose-containing glycoprotein (21). And then in 1982, Kerjaschki et al. identified megalin as an antigen on the rat podocyte membrane and tubular brush border (22) (Table 2). Consistent with previous studies, the pathogenicity of megalin is related to glycosylation (23), and anti-megalin autoantibodies fail to react with megalin in Western blots run under reducing conditions, indicating that autoantibodies recognize the spatial conformation of their epitopes (24). As the rat-specific podocyte target antigen in HN, megalin contains multiple epitopes (25–27), and demonstrates intramolecular epitope spreading during the progression of HN proteinuria (28). Intermolecular epitope spreading has also been observed with the development of autoantibodies to plasminogen, a known ligand of megalin (24). Although megalin is not the pathogenic antigen in human MN, this series of animal studies reveals the possible pathogenesis of human MN, suggesting that autoantibodies bind to podocyte intrinsic antigens to form *in situ* immune complexes in the glomerular subepithelial. Thus, the search for human podocyte-specific target antigens has become the focus of further studies (29).

**Table 2:** Morphological and serological connections between animal and human membranous nephropathy.

	**Human IMN**	**Heymann nephritis**	**Cationic bovine serum albumin model**	**THSD7A-associated Heterologous model**
Immunoglobulin deposition	Mostly IgG4	IgG from rabbit or other sources	Rat or rabbit IgG	IgG from human or rabbit
Complement deposition	C3, C4, and C5b-9	C3, C5b-9	C3, C5b-9	No early complement deposition
Pathogenic antigen	PLA2R1, THSD7A	Megalin	Exogenous cationic bovine serum albumin	THSD7A
Identification in humans	–	None	Identification in early childhood MN	3–5% of IMN
Antibodies in peripheral blood	Anti-PLA2R1 antibody, anti-THSD7A antibody	Anti-Megalin antibody	Rat or rabbit IgG	Anti-THSD7A antibody

*C3, C4, and C5b-9 are all complement components; PLA2R1, The M-type receptor for secretory phospholipase A2; THSD7A, The Thrombospondin type-1 domain-containing 7A*.

### Human Podocyte Antigens

The results from animal model studies have since been validated in humans (Figure 1C). In 2002, Ronco et al. found that the pathogenic antigen in three families of neonatal MN was neutral endopeptidase (NEP) located on the foot process membrane of the podocytes and the brush border of the renal tubules, while the pathogenic antibody derived from NEP deficiency in the mother. This was the first confirmation that the immune complex antigen of human MN could be an intrinsic component of the podocyte membrane (8, 30). More importantly, in 2009 Beck et al. discovered PLA2R1 and its circulating autoantibodies, the first podocyte-targeted antigen-antibody system found in adult IMN (9) (Table 2). The presence of circulating anti-PLA2R1 antibodies can be detected in 70–80% of IMN (31–33). As with megalin, PLA2R1 has multiple antigenic epitopes (34–36), and the epitopes spread as the disease progresses (36). The second target podocyte antigen, THSD7A, was discovered in 2014 by Tomas et al. (10) (Table 2). These findings not only demonstrate that human MN immune complexes form by the same mechanism as seen in HN, but also gives us reason to believe that there are still undiscovered pathogenic podocyte antigen-autoantibody pairs in IMN (37). Other proteins postulated to serve as autoantigens in adult MN include superoxide dismutase (SOD2), aldose reductase (AR), and alfa-enolase. While these proteins typically localize inside the cell, AR, and SOD2 are also expressed on the plasma membrane of podocytes in patients with MN (24, 38, 39). However, the clinical significance and pathogenic role of these cytoplasmic antigens, and the differences between them and the podocyte membrane antigens (PLA2R1 or THSD7A) in pathogenic autoimmune responses has yet to be clearly demonstrated.

### Complement Activation and Podocyte Injury

Another important area of research regarding MN is the mechanism of kidney injury following the formation of immune complexes (Figure 1B). In 1980, Salant et al. found that C3-deficient rats treated with cobra venom factor did not produce proteinuria within 5 days after PHN was induced with sheep anti-rat Fx1A antibody injection (40). Similar results were obtained when HN was established in C6 and C8-deficient rats (41, 42). These findings suggest that complement activation to produce C5b-9 is a key factor in the development of proteinuria in HN. Subsequent studies on experimental MN have further clarified that the pathogenic role of C5b-9 is mainly on podocytes because of its non-dissolving activity. The mechanisms involved include: (1) Inducing podocytes to produce oxygen free radicals; (2) Stimulating podocytes to produce various proteases to cause GBM damage; (3) Influencing a microfilament skeleton structure in podocytes, by separating and redistributing the proteins nephrin and podocin, which are major components of the membrane; (4) Upregulating cyclooxygenase 2 (COX-2) in podocytes, causing damage to the endoplasmic reticulum; (5) Increasing the extracellular matrix by promoting the production of TGF-β by podocytes, leading to GBM thickening and glomerular sclerosis; (6) Promoting podocyte apoptosis and shedding from GBM (43). In addition to the above findings from experimental MN, C5b-9 is also found in the glomeruli and urine of patients with MN, with levels of urine C5b-9 correlating with disease severity and patient prognosis (44, 45), indicating that complement activation is involved in the pathogenesis of human MN. The complement system mainly consists of three early activation pathways (classical pathway, alternative pathway, and lectin pathway), terminal pathway and regulatory system. The formation of C5b-9 is the final step of complement activation (46). Activation of the classical pathway starts with the binding of antibodies to C1q, which can generally be visualized in SMN, and especially lupus-associated MN, by immunofluorescence. However, the subepithelial deposition of immune complexes in IMN mainly consists of IgG4, whose ability to bind C1q is very weak. Indeed, the amount of C1q is very low or undetectable in immune deposits of IMN, indicating that activation of the classical pathway is not the main pathogenic mechanism in IMN (1). Aside from the fact that the presence of IgG1 in early deposits could activate the classical pathway (47), some evidence suggests that the alternative pathway and/or the lectin pathway may be more crucial in the pathogenesis of IMN. Hayashi et al. detected the deposition of mannose-binding lectin (MBL) in glomeruli in IMN, and found that the staining intensity of MBL correlated with the IgG4 staining intensity (48). Bally et al. reported five patients with IMN who had complete MBL deficiency and the complement activation was mainly due to the activity of the alternative pathway (49). Moreover, Luo et al. found that factor B-null mice with established MN did not develop albuminuria or exhibit glomerular deposition of C3c and C5b-9, which suggests that the alternative pathway is necessary in the pathogenicity induced by glomerular subepithelial immune complexes (50).

## Revelation From Other Experimental Membranous Nephropathy

### Exogenous “Planting” Antigen

There is another possibility for the formation of *in situ* immune complexes (Figure 1B). In 1980 and 1981, Batsford et al. and Border et al. created an animal model with the typical pathological lesion of MN by intravenous infusion of cationized exogenous serum proteins (51, 52) (Table 2). The glomerular subepithelial deposition of IgG and C3 can only occur after immunization with a cationized antigen. After immunization with an anionic or neutral antigen, deposition occurs simultaneously in the mesangial area. Furthermore, the proteinuria of an animal that is immunized with cationized antigen is more severe (53, 54). This is due to the negative charge of the glomerular capillary wall, which interacts electrostatically with the cationized antigen resulting in the “planting” of the exogenous antigen. This exogenous antigen then binds to circulating antibodies *in situ* to form the immune complex. In humans, cationic bovine serum albumin (cBSA) is most often the exogenous “planting” antigen found in early childhood MN (55) when the diet of the children is mainly based on milk products. This suggests that some cases of MN may be related to dietary and environmental factors. Moreover, the formation of subepithelial immune complexes in SMN, such as hepatitis B virus-associated MN, is similar (7).

### Other Causes of Podocyte Injury

Complement activation is the crucial mechanism of MN podocyte injury, but it is not the only one. After Beck et al. discovered PLA2R1 in 2009, Tomas et al. found THSD7A, the second adult podocyte antigen, in 2014 (9, 10). THSD7A and PLA2R1 are large transmembrane proteins with multiple domains which are both expressed on podocyte membranes. However, in contrast to PLA2R1, THSD7A is also expressed in mouse podocytes (56). Human anti-THSD7A containing sera can be used to immune-precipitate THSD7A from mouse glomeruli *in vitro* and *in vivo* injections of human anti-THSD7A can specifically bind to murine THSD7A on podocyte foot processes, inducing proteinuria and initiating a histopathological pattern that is typical of MN (57) (Table 2). However, no C3 deposition has been found in the renal tissue of mice shortly following immunization with rabbit anti-THSD7A antibodies, similar to the absence of complement deposition after the injection of purified human anti-THSD7A antibodies (56, 57). These findings indicate that complement activation is not vital in the initiation of podocyte injury and proteinuria in the THSD7A-dependent mouse model of MN. And further, they demonstrate that human and rabbit anti-THSD7A antibodies are directly pathogenic by altering the architecture of podocytes (57). Furthermore, proteinuria also develops in C6-deficient rats with established Heymann nephritis and in C3-deficient mice with established anti-podocyte glomerulonephritis (58–60). In conclusion, the podocyte injury of MN is the result of a complex multifactorial process, especially in IMN where IgG4 deposition predominates, and the mechanism of podocyte injury beyond complement activation needs to be further explored.

## Production and Pathogenesis of Anti-PlA2R1 Antibody

### PLA2R1 and Its Epitopes

**Figure 2:**
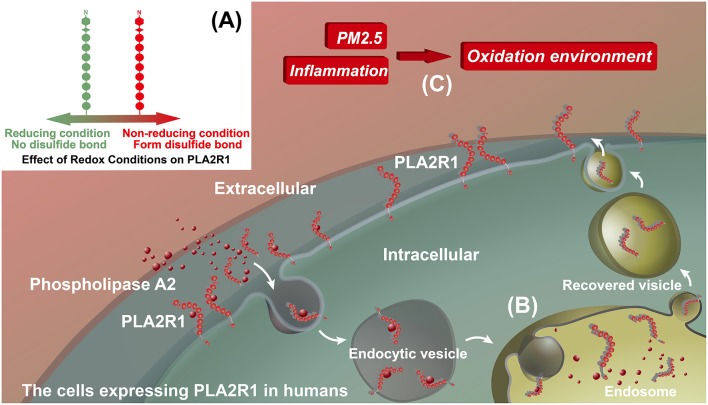
Hypothetical model for the effect of inflammation on PLA2R1. **(A)** Effect of redox conditions on PLA2R1. PLA2R1 without disulfide bond under reducing condition (Green) and with disulfide bond under non-reducing condition (Red). **(B)** The potential endocytic property of human PLA2R1. We hypothesize that the extracellular domain of PLA2R1 binds to phospholipase A2 and is transported into the cell by receptor-mediated endocytosis. This model has yet to be supported by any direct evidence. **(C)** Inflammation, PM2.5, and oxidative microenvironments. We hypothesize that inflammation, including that associated with PM2.5, alters the microenvironment of PLA2R1-expressing cells. PLA2R1 exposed to this oxidative microenvironment may form or retain disulfide bonds resulting the long-term expression of pathogenic epitopes.

PLA2R1 is a type I transmembrane receptor that is a member of the mannose receptor family (61, 62). In general, the mannose receptors have endocytic properties and circulate continuously between the plasma membrane and the endosome, with ~70% of the receptors located inside the cell at steady state (61, 63). PLA2R1 is the specific receptor that promotes the internalization of phospholipase A2 (sPLA2), as has been demonstrated in rat vascular smooth muscle cells, rabbit skeletal muscle cells, and human embryonic kidney (HEK 293) cells transfected with rabbit PLA2R1 and human PLA2R1 (64–66). The exact mechanism by which PLA2R1 mediates PLA2 removal has yet to be determined. Therefore, it may act either as a clearance receptor that inhibits, inactivates and removes sPLA2s from the extracellular milieu, or as a signaling receptor that transduces a sPLA2 cellular signal in a manner independent of the catalytic activity of sPLA2 (62). Several studies have shown that, the physiological role of human PLA2R1 in its expression cells is related to sPLA2s (67–69). However, the interaction of the human PLA2R1 to sPLA2 appears very different from that of other mammals (65), and the endocytic properties of human PLA2R1 has yet to be validated by direct experimental evidence. Thus, we can only propose that, hypothetically, the extracellular domain of human PLA2R1 can bind to sPLA2s and transport it into the cell. In this hypothesis, human PLA2R1 is expressed both intra- and extracellularly (Figure 2B). The higher pH value for extracellular conditions relative to the endosome, vesicle or intracellular environment may result in a more extended conformation of human PLA2R1 (70, 71), which may contribute to the exposure of the human PLA2R1 epitope. The observed epitope spreading may relate to the neutralization of antibodies that bind to the initial epitope (72), which may causes human PLA2R1 to lose its hypothetic endocytic activity and become exposed to the extracellular environment long-term. The PH-dependent conformational change of human PLA2R1 may lead to the exposure of internal domains, which would otherwise not be recognized by the immune system, triggering autoimmune responses to different epitopes. Interestingly, similar to megalin, the binding of IMN patient serum anti-PLA2R1 antibodies to PLA2R1 *in vitro* needs to be carried out under non-reducing conditions, because addition of a reducing agent eliminates the binding of these antibodies (9, 34–36). These facts demonstrate that the epitope(s) to which anti-PLA2R1 antibodies bind is spatial and requires the presence of disulfide bonds in PLA2R1 (Figure 2A). The oxidative extracellular environment may cause PLA2R1 to form or preserve disulfide bonds, resulting in long-term expression of pathogenic epitopes that specifically bind to the anti-PLA2R1 antibodies circulating in the peripheral blood of a patient with IMN (Figure 2C).

### PLA2R1 Exposure

**Figure 3:**
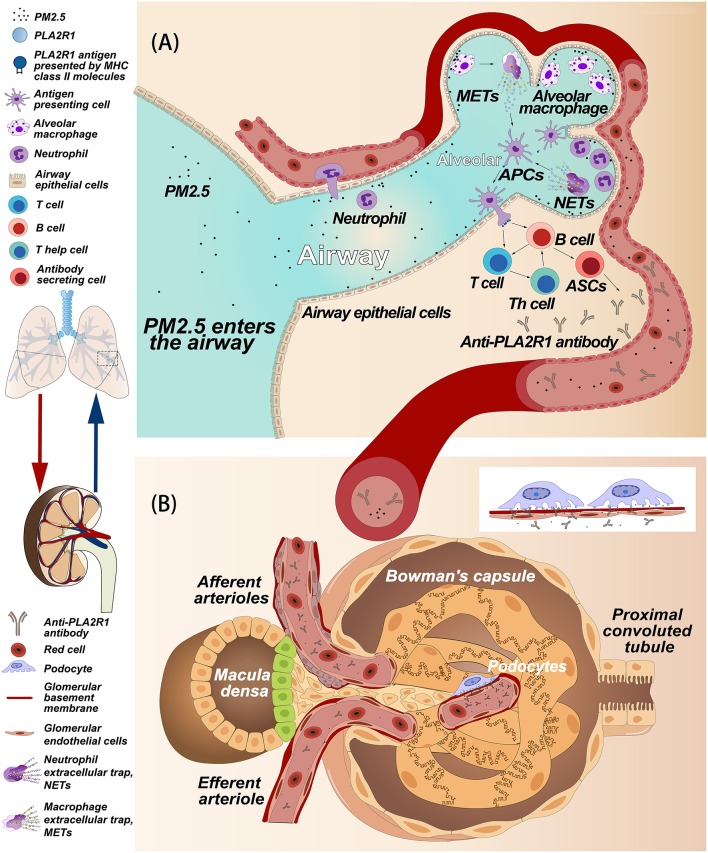
Hypothetical model of the relationship between PM2.5 and membranous nephropathy. **(A)** Hypothesis of how PM2.5 induces anti-PLA2R1 antibody production. PM2.5 in the airways and alveoli causes an inflammatory response involving neutrophils, alveolar macrophages, and airway epithelial cells. We hypothesize that these cells may express PLA2R1, that due to oxidative stress associated with inflammation, may assume a conformation that includes pathogenic epitopes that contribute to the formation of autoantibodies. Alternatively, PLA2R1 may be released into the inflammatory space during the release of extracellular traps. PLA2R1 may then be bound by antigen presenting cells, triggering the humoral immune response, and producing anti-PLA2R1 antibodies. **(B)** The hypothetical process of subepithelial immune complex deposition caused by the anti-PLA2R1 antibody exogenous to glomeruli. Both PM2.5 and anti-PLA2R1 antibodies enter blood vessel and circulate into the glomerular capillaries. The anti-PLA2R1 antibodies penetrate the endothelial cells and glomerular basement membrane (GBM), recognize and bind to naturally-expressed PLA2R1 on podocytes to form the immune complex. These complexes then deposit into the space between podocytes and GBM.

In humans, PLA2R1 is expressed not only in podocytes (9), but also in neutrophils (67), pulmonary macrophages (73), airway epithelial cells, and submucosal epithelial cells (69) (Figure 3A). Podocytes are highly differentiated glomerular capillary epithelial cells whose big cell bodies swell into the crude urine space while the foot processes adhere to the GBM, which is interlaced with the foot processes of adjacent podocytes (74). Normally, the filtration barrier, which is composed of endothelial cells, GBMs and podocytes, only allows water and small molecules to pass freely, while retaining larger molecules such as plasma proteins (75). Even if the glomerulus is easily damaged by the immune system, antigen-presenting cells such as macrophages or dendritic cells remain inaccessible to podocytes if endothelial cells and GBMs are not damaged (75). We hypothesize that podocytes or other cells expressing PLA2R1 may become damaged and release extracellular vesicles (EVs), which can be measured in the urinary tract (76, 77), leading to the onset of autoimmune activity and the development of MN. EVs are small vesicles with membrane proteins on their surface derived from the source cells. Though it has not yet been proved experimentally, cells expressing PLA2R1 may release EVs carrying PLA2R1 on the surface after stimulation. In order to meet the precise specificity required for antigen-antibody binding (78), the epitopes exposed on these non-podocyte cells must be consistent with the PLA2R1 epitope expressed in podocytes, theoretically in a non-reducing environment.

### Inflammation, Oxidative Stress, and PLA2R1

Both neutrophils and pulmonary macrophages are inflammatory cells that can accumulate at the site of inflammation (79, 80). In an inflammatory environment, reactive oxygen species (ROS) are produced in an amount that exceeds the ability of the cell to detoxify itself, leading to an oxidative stress state (81). Under this circumstance, the intracellular environment becomes strongly oxidizing, which results in disulfide bond formation in cytoplasmic proteins (81, 82). Interestingly, there is a significant correlation between the morbidity of MN patients in China and the concentration of PM2.5 (11). Indeed, respirable particulate matter (PM) in polluted air is also associated with various inflammatory lungs diseases (83–85). The occurrence of pulmonary inflammation caused by PM is associated with the production of ROS and oxidative stress (86). Polycyclic aromatic hydrocarbons and transition metals in the PM can directly produce ROS (87, 88). In addition, target cells in the lungs, such as airway epithelial cells, macrophages and neutrophils, can also produce ROS when exposed to PM (87–90). In theory, these cells could aggregate in the airway or alveolar and express PLA2R1 with pathogenic epitopes in response to PM and oxidative stress. PLA2R1 could also then be discharged into the inflammatory space when the neutrophils or macrophages release extracellular traps (91, 92) (Figure 3A). Interestingly, rat PLA2R1 expressed on lymphocytes and granulocytes can be up-regulated by interleukin-1β *in vitro* (93). However, it is unclear whether this is the case with human PLA2R1. Most of the cells involved with inflammation are also capable of presenting antigens (94). Therefore, in theory, PLA2R1 in an inflammatory environment may be more easily recognized by the immune system, thus triggering an autoimmune response (Figure 3A). In addition, PM2.5 exposure in lung could also be considered as a danger signal that induce activation of autoreactive T cells, which also may a trigger be required for induce autoimmune response. However, further studies are needed to explore these hypotheses.

### Risk Genes and Anti-PLA2R1 Antibodies

Two risk alleles (HLA-DQA1 and PLA2R1) were identified in French, Dutch, and British patients with IMN using the genome-wide association study of single nucleotide polymorphism (SNP) Genes (95). Moreover, there is likewise a correlation between HLA-DQA1 and PLA2R1 risk alleles in patients with IMN in the Spanish and Chinese populations (96, 97). Interestingly, anti-PLA2R1 antibodies were detectable in 73% of patients with both PLA2R1 and HLA-DQA1 high-risk genotypes and in none of the patients with both low-risk genotypes (98). HLA-DQA1 produces a receptor protein on antigen presenting cells as part of the major histocompatibility complex, and PLA2R1 produces the specific podocyte target antigen of IMN patients, both of which are related to the triggering of autoimmune responses. Gupta et al. proposed that after PLA2R1 protein has been processed and displayed on the surface of antigen presenting cells as peptides bound to the class II receptor (DQA1) groove, the genetics of DQA1 will shape the amino acid structure of its receptor groove to fit with the peptide sequences available from PLA2R1. Additionally, the genetics of PLA2R1 may control the fragmentation of protein or the level of transcript due to a change in the number of positions of enzyme cut sites, leading to higher levels of peptide (98). The two risk alleles of IMN may be involved in the formation of anti-PLA2R1 antibodies. However, the SNPs identified are common and thus can only partially explain the onset of MN in an individual, even in an individual with multiple risk factors. Therefore, further studies are needed to identifying the triggers or environmental factors that can contribute to MN. It would be especially useful to compare IMN patients with a control population that carries the risk allele (98).

### Pathogenesis of Anti-PLA2R1 Antibodies

In the case where non-podocyte cells initiate the autoimmune response, the process by which the circulating autoantibodies locate, recognize, and bind the target antigens on podocytes is similar to the process seen in the PHN animal model. The exogenous antibodies can penetrate endothelial cells and the GBM to bind with the podocyte target antigen (15) (Figure 3B). However, just as megalin glycosylation and spatial conformation determine its pathogenicity in HN (23), antigenicity of PLA2R1 also requires some additional conditions. When the anti-PLA2R1 antibodies are produced by non-podocyte cell sources in and or enter the circulation, the antibodies will only bind to the podocyte PLA2R1 under non-reducing conditions (9, 34–36). As a cellular transmembrane receptor (61, 62), the extracellular domain of PLA2R1 is in contact with the extracellular environment and thus is most likely to be in the naturally expressed spatial conformation (Figure 2B). PM2.5 also causes early kidney damage through oxidative stress or inflammation (99), which may contribute to the development of non-reducing conditions in the renal microenvironment. Autoantibodies binding to podocyte antigens *in situ* form immune complexes, which result in podocyte injury (1). However, since the immunoglobulins deposited in IMN are mainly IgG4 whose ability to bind C1q is weak, complement may be activated from non-classical pathways (Please refer to the paragraph of “Complement activation and podocyte injury”). Beyond that, antibody binding can also inhibit the normal function of the antigenic protein (72). PLA2R1 may be involved in the adhesion of podocytes to GBM and the serum anti-PLA2R1 antibodies may interfere with adhesion by binding to PLA2R (100), suggesting that serum antibodies binding to podocyte PLA2R1 may cause kidney damage through more than just complement activation.

## Conclusions

Although research on MN has greatly improved quality of life in the patients, there are still many unsolved mysteries (Table 3). In this review, we have mainly discussed the pathogenesis of PLA2R1-associated MN, and proposed some hypotheses based on the available research. We believe that further research into these questions will be beneficial to the clinical treatment of IMN patients by further revealing mechanisms behind the development of pathogenic antigens and antibodies and searching for treatments that prevent or inhibit the resultant kidney damage.

**Table 3:** Known and unknown about the pathogenesis of membranous nephropathy.

	**Known**	**Unknown**
Membranous nephropathy	Changes to the GBM caused by IC deposition.	In addition to complement activation, what are the causes of proteinuria?
	(1) Circulating ICs deposition; (2) *in situ* ICs deposition: the podocyte antigen or the foreign “pre-planted” antigen.	What are the physiological functions of PLA2R1 and THSD7A?
	IC-associated complement activation leads to proteinuria.	How is complement activated in IMN?
	HLA-DQA1 and PLA2R1 are risk alleles in IMN.	What role do risk alleles play in pathogenesis?
	The incidence of MN is related to environmental and diet.	How do environment and diet affect MN patient population?
PLA2R1-associated MN	PLA2R1 is expressed in multiple places in the human body.	Where the PLA2R1 expose? How and the relevant influencing factors?
	The anti-PLA2R1 antibody is the serum marker.	How is the humoral immune response initiated?
	PLA2R1 epitope spread as the disease progresses.	How does epitope spreading occur? Why is it associated with disease progression?
THSD7A-associated MN	THSD7A-associated MN is significantly associated with malignancies.	What is the role of THSD7A in membranous nephropathy and malignancies?

*GBM, glomerular basement membrane; ICs, Immune complexes*.

## Author Contributions

WenL collected most of the material for reviewing and wrote the main part of the review. CG and HD collected the rest of the material for reviewing. YZ, ZD, and YG wrote the rest part of the review. FL, ZZ, and ZL made the figures and tables. WeiL, JS, QL, and BL discusses and modifies the content of the review article.

### Conflict of Interest Statement

The authors declare that the research was conducted in the absence of any commercial or financial relationships that could be construed as a potential conflict of interest.
